# Hand hygiene during facility-based childbirth in Cambodia: a theory-driven, mixed-methods observational study

**DOI:** 10.1186/s12884-021-03901-7

**Published:** 2021-06-17

**Authors:** Yolisa Nalule, Helen Buxton, Por Ir, Supheap Leang, Alison Macintyre, Ponnary Pors, Channa Samol, Robert Dreibelbis

**Affiliations:** 1 Disease Control Department, London School of Hygiene and Tropical Medicine, London, WC1E 7HT UK; 2 Division of Psychiatry, University College London, London, W1T 7BN UK; 3 National Institute of Public Health, Phnom Penh, Cambodia; 4 WaterAid Australia, Melbourne, Australia; 5 WaterAid Cambodia, Phnom Penh, Cambodia

**Keywords:** Hand hygiene, Maternal infection, Intervention design, Formative research, Infection prevention and control

## Abstract

**Background:**

Despite current efforts to improve hand hygiene in health care facilities, compliance among birth attendants remains low. Current improvement strategies are inadequate, largely focusing on a limited set of known behavioural determinants or addressing hand hygiene as part of a generalized set of hygiene behaviours. To inform the design of a facility –based hand hygiene behaviour change intervention in Kampong Chhnang, Cambodia, a theory-driven formative research study was conducted to investigate the context specific behaviours and determinants of handwashing during labour and delivery among birth attendants.

**Methods:**

This formative mixed-methods research followed a sequential explanatory design and was conducted across eight healthcare facilities. The hand hygiene practices of all birth attendants present during the labour and delivery of 45 women were directly observed and compliance with hand hygiene protocols assessed in analysis. Semi-structured, interactive interviews were subsequently conducted with 20 key healthcare workers to explore the corresponding cognitive, emotional, and environmental drivers of hand hygiene behaviours.

**Results:**

Birth attendants’ compliance with hand hygiene protocol was 18% prior to performing labour, delivery and newborn aftercare procedures. Hand hygiene compliance did not differ by facility type or attendants’ qualification, but differed by shift with adequate hand hygiene less likely to be observed during the night shift (*p* = 0.03). The midwives’ hand hygiene practices were influenced by cognitive, psychological, environmental and contextual factors including habits, gloving norms, time, workload, inadequate knowledge and infection risk perception.

**Conclusion:**

The resulting insights from formative research suggest a multi-component improvement intervention that addresses the different key behaviour determinants to be designed for the labour and delivery room. A combination of disruption of the physical environment via nudges and cues, participatory education to the midwives and the promotion of new norms using social influence and affiliation may increase the birth attendants’ hand hygiene compliance in our study settings.

**Supplementary Information:**

The online version contains supplementary material available at 10.1186/s12884-021-03901-7.

## Background

Contaminated hands of the health care worker (HCW) is the main transmission pathway via which many healthcare associated infections (HCAI) are spread to patients [1–3]. Healthcare associated infections affect 15% of patients in low and middle- income countries (LMIC) [4]. Adequate HCW hand hygiene during the peri-natal period is particularly critical for the prevention of maternal [5, 6] and neonatal infections [7]. Globally, 10% of maternal deaths [8] and 11- 19% of neonatal deaths [9] have been attributed to infections, most of which are acquired during labour, delivery and the first week of a newborn’s life [8, 10, 11]. Despite the implementation of various hand hygiene promotion strategies in healthcare facilities (HCF) [12, 13], hand hygiene compliance in both maternal [14] and newborn care [15–17] remains low.

Hand hygiene is a behaviour influenced by multiple factors [4, 18] and interventions to improve hand hygiene behaviours are more effective when they target the context- and behaviour-specific determinants of hand hygiene outcomes [19–21]. However, the current understanding of the drivers of hand hygiene behaviours specific to labour and delivery is limited [12, 13]. Hand hygiene improvement strategies targeting HCW often result in small or moderate effects [12, 22] and are often only short-term [13, 23]. Current approaches to improving hand hygiene practices among HCW largely focus on a limited set of known behavioural determinants (knowledge, skills, and physical opportunity), provide limited information on the specific determinants interventions target, or address hand hygiene as part of a generalized set of hygiene behaviours [18, 24].

In Cambodia, 83% of all births take place in the HCF [25]. Hand hygiene among midwives in Cambodia is low [26] and while there is limited data on the attributable burden of HCAI on maternal and newborn outcomes, studies have identified existing gaps in infection prevention and control (IPC) within HCF, potentially putting a large number of mothers and newborns at increased HCAI risk [26–28]. A 2016 cross-sectional survey of 117 public HCF across 5 provinces found that while 92% of HCF had at least one functioning hand washing facility (HWF), only 3% of HCF met the basic service level for hand hygiene [28]. In 2017, the Ministry of Health (MOH) of the Royal Government of Cambodia launched its revised IPC guidelines for HCF [29] and in 2018, released the first national guidelines for Water, Sanitation and Hygiene (WASH) in HCF [30]. Together, these guidelines emphasise the provision of services and HCW training on hand hygiene practices that align with the WHO recommended Five Moments for Hand Hygiene approach for healthcare workers during patient care [3]. Hand hygiene and IPC guidelines specific to childbirth are integrated within the national Maternal and Child Health guidelines [31] and midwives receive further training in clean birthing practices, and provision of hygienic post-partum and postnatal care. Hand hygiene behaviour change and promotion strategies however are not explicitly addressed within all these guidelines.

To this end, mixed-methods, theory-driven formative research was carried out in Kampong Chhnang province, Cambodia, to document the hygienic conditions of the HCF delivery units, observe hygiene practices during childbirth and to explore the determinants of observed hand hygiene practices among HCW. This study is part of a larger project—Changing Hygiene Around Maternal Priorities (CHAMP) to design and test a hand hygiene intervention targeted at multiple stages along the continuum of care, from childbirth to return to the home environment. The findings from this formative research study were used to inform the development of intervention components aimed at improving hand hygiene during the labour and delivery stage. Formative research findings related to the other stages along the care continuum—facility and home post-natal care, will be reported in forthcoming publications.

## Methods

### Study setting and design

The formative research study followed a sequential explanatory mixed-methods design [32] and was guided by the Behaviour Centred Design (BCD) approach, which combines theory-based, ecological –evolutionary understanding of human behaviors with a systematic process for intervention development and evaluations [33, 34]. The BCD formative research process employs a checklist of behavioural determinants classified into 4 components (body, brain, environment, behaviour setting) and a range of formative research tools to comprehensively investigate and identify key practices, targets, behavioural determinants and pathways to change within a specific context. Table 1 provides definitions of each BCD determinant adapted for handwashing behaviour [35].

**Table 1:** Adapted BCD checklist of handwashing behavioural components and determinants [35]

BCD component	Determinant	Definition of each determinant adapted to handwashing
**Brain**	Executive Brain	• The extent to which knowledge of handwashing behaviour and its benefits affects handwashing intentions and plans, and eventually performance of the behaviour
	Motivated Brain	• The goal-related drivers of behaviour. Motives for handwashing can include (but is not limited to) disgust (the desire to avoid cues to sources of infection), affiliation (the desire to fit in with others) and nurture (the desire to care for your child)
	Reactive Brain	• The extent to which handwashing can be automatically triggered based on past experience and repetition
	Discounts	• The perceived time, effort and costs of washing hands with soap as compared to other courses of action
**Body**	Characteristics	• Socio-demographic characteristics that may affect handwashing, including gender, wealth, age, education and employment
	Senses	• The sensory perceptions that may cue handwashing behaviour or be experienced during or after handwashing
	Capabilities	• Whether an individual has the skills required to wash their hands with soap • Whether an individual perceives themselves to be able and willing to actually wash their hands at the times required
**Behaviour settings**	Stage	• The design and set up of the specific physical spaces where handwashing behaviour takes place
	Infrastructure	• Durable infrastructure associated with handwashing such as water supply systems, sanitation, kitchen facilities and handwashing facilities
	Props	• The value, characteristics, usability, ownership and accessibility of soap and other objects used for handwashing
	Roles	• The ways in which an individual’s role, identity or responsibilities influence their handwashing practices
	Routine	• The sequence of behaviours regularly performed in association with handwashing
	Norms	• The extent to which an individual’s handwashing practice is influenced by their perception of normative setting-specific rules. This includes an individual’s perception of whether handwashing is commonly practiced in their community (descriptive norm); whether handwashing is part of their role and their normal behaviour (personal norm); whether handwashing is socially approved of (injunctive norm); and whether handwashing is practiced by their ‘valued others’ (subjective norm)
**Environment**	Physical environment	• Factors in the physical or built environment including climate and geography
	Biological Environment	• Factors associated with an individual’s interaction within their biological environment
	Social Environment	• The structure of an individual’s social environment, including how they interact with it and perceive themselves within it
**External context**	Political and historical context	• The historical and cultural events that have shaped current perceptions and practices of handwashing. The extent to which handwashing-related policies or local and national leadership on handwashing issues, shape handwashing perceptions and practices at the individual level

The study was conducted in Kampong Chhnang Province located in the central part of Cambodia. Kampong Chhnang has a total population of approximately half a million people, 80% living in the rural areas [36]. There are 42 primary health centres (PHC) in the province that provide outpatient consultations, emergency care and minor surgery; care for under-five children and pregnant women including antenatal care and normal delivery and family planning services. These facilities are supported by two referral hospitals (RH) that provide referral consultations, general hospitalization for adults and pediatrics, medical and surgical emergencies, including complicated delivery and tuberculosis cases with laboratory, ultrasound and x-ray.

The study was carried out in six PHC and two RH. We purposively selected the six PHC with the highest number of monthly deliveries to ensure a sufficient number of observations. All two RH located in Kampong Chhnang Province were included in the study. Observational data collection occurred in 2019 from February to July and qualitative data were collected over 2 weeks in September 2019.

### Quantitative methods

#### Data collection

##### Observational assessments

A structured facility walkthrough and needs assessment survey, adapted from standard tools; WHO WASHFIT [37] and SoapBOX WASH & Clean Toolkit [38], were conducted in the delivery rooms and post-natal wards of each of the eight facilities. The facility needs assessment survey was conducted with the member of staff designated in charge of the maternity ward on the first day of the observations.

##### Structured observations

Hand hygiene practices were assessed through structured observations of women during uncomplicated vaginal births. The study population consisted of women in labour admitted to any of the selected HCF during a 14-day observation period and the corresponding HCW, ancillary workers and family members involved in the care of the woman and the newborn.

Eligible women for recruitment were those who presented to the HCF for delivery prior to entering the second stage of labour and were not already in excess pain or distress. The recruitment was done in a private area away from other patients and HCF staff and women were encouraged to have someone else with them to hear more about the study. The purpose of the study was explained to all participants who were approached and written consent obtained. In the case of illiterate participants, verbal consent was obtained in addition to a witness signature. Participants were informed that the aim of the study was to observe care giving practices during childbirth and post-natal care in order to make recommendations to improve the quality of care for women and newborns. The explicit mention of handwashing was avoided to minimise reactivity. Participants were informed that their consent was voluntary, they could request to stop participating, or to take a break at any time and that their consent or withdrawal would have no impact on services provided and would not harm or benefit themselves or their baby. The data collector discussed and agreed with consenting participants verbal or non-verbal cues that they could use to pause or terminate the observations. Patients considered by clinical staff to have a complicated labour or delivery were excluded from the study. Inclusion and exclusion criteria are expanded upon in additional information (Additional file 1).

In the six PHC, women were recruited until either five births per facility had been observed or the 14-day observation period ended. In the RH, as many women as possible were recruited and observed over a period of 14 continuous days. The sample size was considered sufficient for the exploratory nature of the study.

Observations began when the consenting woman was admitted to the facility and the first vaginal examination occurred, and observations terminated either after 6 h or when the woman and the newborn were discharged from the delivery room, whichever came first. All women were given a 15-min break from observations every 2 h. Observations were terminated prior to the pre-defined endpoints if the mother or the attending clinical health worker or caregiver requested the observation be terminated, there was an emergency or complication for mother or newborn, or the newborn was transferred to a different ward/area and became separated from the mother (e.g. child is transferred to a neonatal intensive care unit).

Observations were recorded on tablets using a structured tool, pre-coded with key events using the Open Data Kit software. Data collectors positioned themselves in an unobtrusive location in the delivery room and chronologically recorded key events of all individuals present during childbirth and provided qualitative notes when necessary. Key events included in the observation tool were selected based on literature reviews and previous observations of hygiene during childbirth [17] and included: HCW handwashing and gloving practices; all contact during maternal and newborn care with an emphasis on any aseptic procedures; hand contact with objects and surfaces; and location of mother and newborn.

A team of qualified midwives were trained as study observers. The training included a review of medical processes, content procedures, ethical research practices, simulated birth observation practice, role-play and video sessions to ensure consistent and reliable use of the data collection tools and protocols. All observation tools and protocols were piloted in the field prior to data collection in two non-study HCF in Kampong Chhnang where the midwives observed six deliveries and conducted two facility structured walkthroughs. The tools were iteratively refined during the 7-day training period. Study observers worked in pairs through-out the pilot period to increase interrater reliability. Given the small sample size, rather than use statistical techniques to formally test reliability, observers worked independently to gather data and then compared results with their pair at the end of the collection period. Any discrepancies would be followed up with the study manager for clarification and discussion. Observation data was visually examined at the end of each day to look for discrepancies, and if found, certain procedures were modelled in training so that study observers could practice data collection. Observers returning richer observation data were paired with those returning sparser data to mentor each other. A further 3-day refresher training was conducted prior to the data collection at the RH.

#### Data analysis

All quantitative data was analysed using StataSE 15 (Stata Corp, College Station, TX, USA). Qualitative notes recorded during the observations were reviewed and where applicable, recoded using STATA.

Data were described dynamically using the analysis process adapted from Buxton et al. [17]. We identified procedures conducted during labour, delivery or newborn aftercare that required aseptic technique—hands washed with soap and gloves worn, avoiding recontamination of both washed and gloved hands [3]. The identified aseptic procedures were then bundled into ‘flows’ – a concept adapted from Gon et al. [39] which describes a sequence of aseptic procedures conducted consecutively without hand hygiene necessary in between each one, provided the HCW has avoided invalidation of aseptic technique (Table 2). The beginning of a flow and the procedures included within it were determined by a combination of data output from the direct observations, WHO guidelines [40–43] and existing literature [39].

**Table 2:** Definition and description of flows and aseptic procedures used for the analysis

**Flow**	**Labour**	**Delivery**	**Newborn Aftercare**
**Description**	Vaginal examinations	Approximated to begin as birth attendants were donning full personal protective equipment and concluded after the delivery of the placenta^a^	Approximated to begin when contact with the newborn is made to separate from the mother for initial weighing & body inspection and concluded when the newborn is brought back to the mother
		Suturing of the perineum^b^	
**Aseptic procedures of interest**	Vaginal wiping	Artificial rupture of membranes	Newborn full body inspection
	Fingers in vagina	Episiotomy	Umbilical cord examination
		Assisted delivery using forceps or vacuum	Newborn immunisation
		Newborn delivered	
		Newborn cleaned	
		Controlled cord traction	
		Placenta delivered including manual removal	
		Sweeping of uterus post-delivery	
		Vaginal & rectal examination post- delivery	
		Perineum sutured	
		Vaginal & rectal examination post- suturing	
		Vaginal wiping	

^a^Any procedures that were done on the newborn baby prior to its first separation from the mother for weighing and body examination were considered part of the delivery flow

^b^A separate delivery flow would be indicated if suturing of the perineum was performed

Proactive and reactive hand hygiene opportunities around the three flows were identified, based on the WHO five moments of hand hygiene [3, 43]. Each flow was counted as one hand hygiene opportunity and additional hand hygiene opportunities arose only if aseptic technique was invalidated within the flow. Examples of activities that would invalidate aseptic technique within the flow included HCW exiting the room or HCW contact with surfaces such as the delivery bed and trolley, non-sterile equipment and materials, clinical waste, body soiling (faeces, blood and other bodily fluids) and other individuals during a flow. For both the labour and delivery flows, HCW contact with the mother in the *patient zone* [3, 43] was not counted as a hand hygiene opportunity. We defined the mother’s patient zone as the area around her lower abdomen, vagina and upper thighs [39]. The newborn was also considered a part of the mother’s patient zone until its first separation from the mother, for the initial inspection and assessments.

For each individual, their corresponding hand hygiene actions around each hand hygiene opportunity were coded into three categories for the analysis as described below (Table 3):

**Table 3:** Hand hygiene categories used in analysis

**Hygiene Category**	**Hand hygiene action**
Adequate hand hygiene	Hands washed with soap and new gloves (multiple or single) worn at each hand hygiene opportunity, no potential recontamination of gloved and/or washed hands observed
Inadequate hand hygiene	Gloves (multiple or single) are changed, no handwashing with soap in between glove changing
Aseptic technique invalidated	No hand hygiene actions taken at observed hand hygiene opportunity

All individuals started off automatically assigned to the “aseptic technique invalidated” hand hygiene category, and would change categories throughout the entire observation period based on their hand hygiene actions at any given time.

Descriptive statistics were used to calculate the proportion of flows that were initiated under each hand hygiene category and the proportion of observed hand hygiene practices in all flows combined by facility type (RH vs PHC), provider type (doctor vs. midwife) and shift (morning vs. evening vs night) under each hand hygiene category. *SomersD,* clustered by facility, was used to calculate association between provider type, health facility type, and shift variables and initiating flows with 1) invalidated hand hygiene (aseptic technique invalidated = 1; inadequate hand hygiene + adequate hand hygiene = 0) and adequate hand hygiene (adequate hand hygiene = 1; aseptic technique invalidated + inadequate hand hygiene = 0). All analyses were adjusted for repeated observations of the same provider within each observation period.

### Qualitative methods

#### Data collection

Findings from the structured observations were reviewed by project stakeholders during a 2-day framing workshop (22 – 23 August, 2019, Phnom Penh), including MOH representatives at national, provincial and district levels, HCF directors and development partners. The identified key behaviour of interest for in-depth qualitative investigation specific to the delivery unit was hand hygiene and glove use at critical moments during childbirth among healthcare workers (HCW), with the midwife as the key target for behaviour change.

Over a period of 2 weeks in September 2019, semi-structured interviews were conducted with midwives at selected HCF. The sample size was based on the anticipated number required to reach theoretical saturation while still capturing diversity within and between facilities and respondent categories. Within the context of a single semi-structured interview, additional BCD formative research tools [34] were completed to actively engage midwives and capture data on their routines, norms, motives, emotional and physical drivers of identified behaviours. Descriptions of the data collection tools are detailed (Additional file 2).

All interviews were conducted in Khmer by two teams of two female interviewers who had prior experience in qualitative data collection. Qualitative tools were tested and refined during a 3-day training in a non-participating facility prior to data collection. All interviews were audio recorded and free form notes taken. For each of the data collection activities, responses and ranked sequences were recorded on data capture forms and pictures of completed sets of cards taken. Immediately following data collection, written summaries were prepared on a semi-structured data capture form. At the end of each day, debriefing sessions were conducted and tools were iteratively refined and adapted accordingly.

#### Data analysis

Qualitative data analysis was focused primarily on understanding the drivers of observed hand hygiene behaviour and completed in two phases using Microsoft Word and Excel (Redmond, Washington) by two team members; one who was fluent in the Khmer language. Preliminary data (field notes, written response summaries and any salient findings from daily debriefs) were entered into a spreadsheet and organised by data collection method and activity. Findings were discussed and summarised by study team members and summary notes and audio recordings were consulted for clarity or further exploration as needed. In the second phase of analysis, all data (spreadsheet and original audio recordings) were reviewed again and relevant findings summarised against the pre-defined BCD categories of behavioural determinants [33]. Findings were again reviewed and discussed among team members throughout the analysis process to ensure consistent understanding and interpretation of data.

## Results

### Facility characteristics

All facilities had a designated maternity ward with specific room/s within the ward for deliveries. The delivery rooms had similar physical layouts with designated areas for labour and delivery, newborn assessment, waste disposal, and storage of personal protective equipment (PPE) and birth kits.

All eight labour and delivery wards had functional handwashing facilities with soap, alcohol based hand rub (ABHR) and gloves (clean and sterile). Water was available via a sink with a connected tap in all delivery wards except for one RH which was undergoing construction. All handwashing facilities in the delivery room were visibly clean, accessible and had available and visibly clean hand drying materials. Hand hygiene posters were present, visible and displayed at the handwashing facilities in all but one delivery room.

### Structured observations

#### Participant information

A total of 45 mothers were observed; 22 from the PHC and 23 from the RH. Mothers from the PHC and RH had similar characteristics with a mean age of 28 (21 – 40) and had an average of 2 (0 – 6) previous live births. The average travel time to the HCF was 19 min (5 – 40).

The average number of facility birth attendants present per delivery was 2 (1—5). The midwife was the most common birth attendant, present for 100% of all births. 18% of the deliveries were attended to by only one birth attendant.

#### Labour flow

A total of 95 labour flows were observed, for an average of 2 (range: 0 – 10) vaginal examinations per woman. Birth attendants initiated only 22% of the labour flows with adequate hand hygiene (Table 4).

**Table 4:** Hygiene risk categories prior to all flows combined by provider type, facility type and work shift

	**n**	**Adequate**	**Inadequate**	**Aseptic Technique Invalidated**	**Somers’ D clustered by facility; *p*-value (Confidence interval)**
**Flow type**					
Labour	95	21 (22%)	52 (55%)	22 (23%)	Ref
Delivery	102	19 (17%)	36 (35%)	47 (46%)	0.25; *p* = 0.00 (0.15 – 0.35)
Newborn aftercare	54	4 (7.4%)	11 (20.4%)	39 (72%)	0.46; *p* = 0.00 (0.34 – 0.59)
All flows	251	44 (18%)	99 (39%)	108 (43%)	
**Provider type**					
Sec. Midwife	145	27 (19%)	54 (37%)	64 (44%)	Ref
Primary Midwife	93	15 (16%)	39 (42%)	39 (42%)	-0.05; *p* = 0.8 (-0.34 – 0.25)
Intern	3	1 (33%)	1 (33%)	1 (33%)	0.02; *p* = 0.14 (-0.01 – 0.04)
Doctor + Nurse	5	0 (0%)	3 (60%)	2 (40%)	-0.05; *p* = 0.206 (-0.12 – 0.03)
**Facility type**					
Primary Health Centre	137	17 (13%)	58 (42%)	62 (45%)	Ref
Referral Hospital	110	26 (24%)	39 (35%)	45 (41%)	0.24; *p* = 0.20 (-0.12 – 0.60)
**Shift**					
Morning	109	24 (22%)	35 (32%)	50 (46%)	Ref
Afternoon	49	10 (20%)	18 (37%)	21 (43%)	-0.03; *p* = 0.59 (-0.16 –0.09)
Overnight	89	9 (10%)	44 (49%)	36 (41%)	-0.25; *p* = 0.03 (-0.47 – -0.02)

Over half of observed labour flows (55%) were initiated under inadequate hand hygiene and 23% initiated when aseptic technique had been invalidated.

#### Delivery flow

Birth attendants initiated the majority (46%) of the delivery flows (*n* = 102) when aseptic technique had been invalidated, 35% when hand hygiene was inadequate and only 19% under adequate hand hygiene (Table 4).

**Fig. 1:**
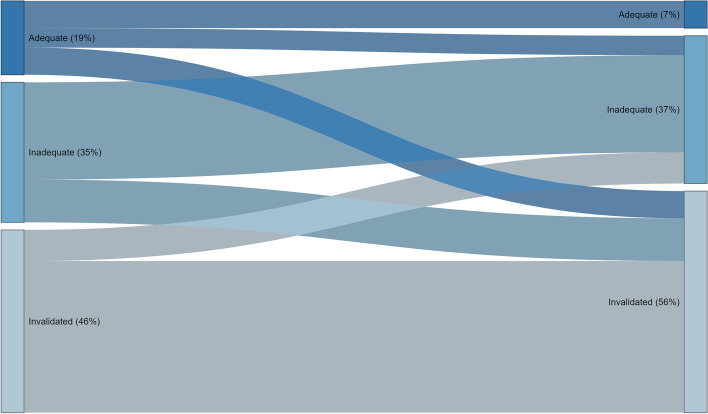
Maintenance of hand hygiene during delivery flow

The proportion of flows that maintained, dropped or improved a hand hygiene category by the end of the delivery flow is represented graphically in Fig. 1. Only 7 of the 19 (37%) flows initiated under adequate hand hygiene maintained this status throughout the delivery flow; 5 of 19 (26%) dropped to inadequate hand hygiene, all of which were glove changes without intermediary handwashing during manual removal of placenta procedures.

7 of 19 of flows (37%) that were initiated under adequate hand hygiene had dropped to invalidated aseptic technique at the end of the delivery flow. The most common reason for invalidation of aseptic technique was donning other PPE items such as non-sterile apron and boots after having already conducted adequate hand hygiene.

The majority of delivery flows that started under inadequate hand hygiene (69%) and those where aseptic technique had been invalidated (83%) maintained those categories throughout the flow. Wiping off faecal and bloody matter from the perineum, floor and trolleys between procedures without subsequent hand hygiene action was the most common observed activity for invalidated aseptic technique. There were only limited improvements in hand hygiene during delivery flows, however these improvements were all inadequate (only gloves changed) and did not fully adhere to hand hygiene protocol.

#### Newborn aftercare flow

**Fig. 2:**
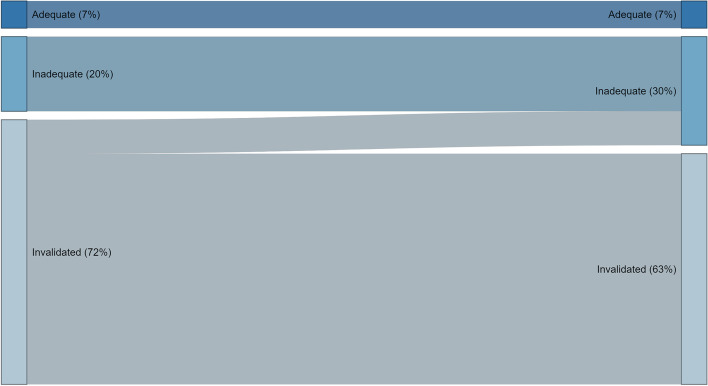
Maintenance of hand hygiene during newborn flow

72.5% of all newborn aftercare flows (*N* = 54) were initiated when aseptic technique was invalidated, 20.5% with inadequate hand hygiene and 7% with adequate hand hygiene (Table 4). All the newborn care flows initiated under adequate or inadequate hand hygiene maintained these categories throughout (Fig. 2).

Only 13% (5/39) of newborn aftercare flows that were initiated when aseptic technique was invalidated improved over the course of the aftercare flow. All observed improvements in hand hygiene occurred just prior to immunisation of the baby when gloves would be donned without handwashing with soap.

Invalidation of hygiene protocol was more likely as the birth process progressed (Table 4). Both delivery and newborn aftercare flows were more likely to be initiated with invalidated hand hygiene compared to labour flows [Delivery: Somers’ D = 0.25, *p* = 0.00; Newborn: Somers’ D = 0.46, *p* = 0.00] and invalidated hygiene practices also more likely to be initiated under newborn aftercare flows than delivery flows [Newborn: Somers’ D = 0.23; *p* = 0.00; VE: Somers’ D = -0.24, *p* = 0.00, data not shown]. When all flows are combined, compliance did not differ by facility type (RH vs PHC) or birth attendant qualification and differed significantly by working shifts. Compared to the morning shift (6:00 – 12:00), adequate hand hygiene was less likely to be practiced during the overnight shift (18:00 – 6:00) [Overnight: Somers’ D = -0.25; *p* = 0.03] but did not differ significantly with the afternoon shift (12:00 – 18:00) [Afternoon: Somers’ D = -0.03; *p* = 0.59].

### Qualitative results

#### Participant information

Qualitative data was collected from 4 HCF; 3 PHC and 1 RH. A total of 20 key healthcare workers were interviewed across the HCF. Not all participants completed all data collection activities and the exact activities depended on the specific respondent or the time available for the interviews.

Interviews with midwives revealed insights into behavioural determinants that promoted or constrained the performance of adequate hand hygiene practices. The findings below are organised according to the key components of the BCD approach (Table 1). Within each component, the identified relevant key behavioural determinants are summarised.

#### Brain + body

##### Knowledge and risk perception

Overall, midwives demonstrated adequate awareness and general understanding of the importance of adequate hand hygiene and gloving practice and their associated link to infection transmission. However, knowledge around avoiding glove and clean hand recontamination including the correct hand hygiene protocol following recontamination was limited. For example, midwives correctly identified washing hands with soap and wearing new gloves before conducting vaginal examinations during labour as proper protocol but did not consider it necessary to do so when they used their gloved hands to wipe a blood spot off a surface during delivery.

Infection risk perception around newborn aftercare was low. With the exception of cord care, midwives considered all other newborn aftercare events in the delivery room including the initial assessment of the newborn following birth, as very low infection transmission risk events. This corresponded to the structured observations where gloves were almost always changed prior to cutting the cord but very few hand hygiene actions observed around other newborn aftercare events.

Refresher midwifery training was irregular and usually externally held with limited opportunities for attendance. Typically, only one midwife and facility director were facilitated to attend external trainings. The extent to which hand hygiene was covered varied by training with some being hand hygiene specific while others included hand hygiene within larger training on general maternal and child health. Most of the midwives could not remember the last time they had attended a training and recalled their formal midwifery education program as the last time anyone had provided formal hand hygiene and IPC training. Midwives relied on knowledge sharing meetings held by the few HCWs who attended the external formal trainings and informally through observing and following peers’ practices, particularly those of the senior midwives, during deliveries.

##### Senses and motives

Midwives pointed to the visibility of potential contaminants serving as their cue for reactive hand hygiene. The presence or contact with soiling, particularly faecal matter, was considered disgusting by all midwives, triggering hand washing with soap and glove changes. However, in most of the structured observations, the presence of visible soiling would typically trigger cleaning/wiping actions such as wiping of soiling on the perineum, delivery surface or floor, further contaminating the gloved hands, but no subsequent hand or glove hygiene actions would be practiced.

##### Discounts

Midwives often reported leaving out or forgetting hand hygiene steps particularly when they were under high pressure situations such as birth complications, quick labours, solo shifts and multiple women in labour. Shortage of staff particularly during the night shift was also a commonly reported challenge. In these situations, forgetting to wash hands with soap and change gloves in between or using the same pair of gloves from start to finish was considered common, although in other cases midwives employed these as deliberately as time saving practices. Multiple gloving was also considered a common and acceptable time saving practice.

#### Behaviour settings

##### Roles and responsibilities

Across all HCF, midwives had a strong sense of ownership towards the delivery room and assumed all the responsibility for all activities that took place in the room including its general appearance. In addition to performing deliveries, midwives’ responsibilities were to: ensure the availability of clean and sterilised PPE, delivery equipment and hand hygiene materials for each delivery; ensure that hand hygiene and glove use protocols are followed during delivery; maintain a clean and odourless delivery room after each delivery; and, ensure regular cleaning, sterilising and proper waste management.

Prior to each delivery, the roles were decided by the midwives, with one midwife primarily responsible for care of the mother during labour and delivery including cutting the cord, and the another midwife taking on newborn aftercare such as physical inspection of the baby, taking weight and measurements and supporting the breastfeeding initiation. These roles switched with each delivery depending on discussion and agreement with the midwives available on shift. All birth attendants present in the room assisted and supported each other throughout the delivery process.

##### Props and infrastructure

Similar to findings from the structured observations, midwives reported the regular availability of functioning and accessible handwashing facilities. In the rare event of a lack of water supply, midwives reported tasking the woman’s relatives to bring enough water into the delivery room for the duration of the delivery. Similarly, running out of gloves, handwashing soap and alcohol rub was reported as uncommon. The stock-out reporting process was simple and well understood, and midwives were responsible for monitoring the stock of these items daily and reporting to the facility director or accountant directly or during the regular staff meetings whenever supplies were low.

In response to what they did or would do when the rare unanticipated stock out did occur, midwives reported compromising hygiene practices in the interim such as using non-sterile gloves to carry out procedures until sterile gloves were replaced or limiting the frequency of glove changes per delivery. In other cases, midwives bought missing materials using their own money and were reimbursed later.

##### Routine

Multiple gloving and the subsequent layer-by-layer removal during the delivery flow was the accepted standard of practice in facilities and integrated into the standard caregiving routines. Wearing only one pair of gloves was unanimously considered a serious infection risk for the midwife and mother, and a midwife would only likely do this if there was a glove shortage. Midwives typically wore two or more pairs of gloves at the beginning of delivery consisting of one pair of clean gloves and one or more pairs of sterile gloves. ‘Changing gloves’ was described by the midwives as removing the top pair and either immediately proceeding with the delivery process with the gloves underneath or donning an additional layer of new sterile gloves prior to proceeding. In line with direct observations, midwives reported routinely removing their outermost gloves at two time points; prior to cord cutting and before delivery of the placenta. Handwashing with soap was never conducted in between these removals as the midwife considered the gloves underneath to still be sterile.

#### Environment

##### Social environment

Within the delivery room, the midwives, superseded only by doctors, were typically at the top of the social hierarchy. In this setting, midwives were highly respected, listened to and were considered authoritative figures by the nurses, patients and the visitors. The social environment between the midwives was cohesive and reported to be generally very supportive and with mutual respect for each other regardless of rank. Midwives perceived their hand hygiene behaviours as easier to change compared to those of the cleaners and visitors because of their strong social relationship crediting the presence of strong systems of support, knowledge sharing and accountability among each other, with everyone being open to correction, and willing to follow and learn from one another.

Maintenance of the social standing of a fellow midwife was typically prioritised over the immediate danger of potential infection transmission to the mother. For the majority of midwives, correction of a colleague in front of a patient was considered an upset to that social order. Midwives also perceived public correction to lead to loss of trust/confidence and respect between the mother and the HCW. As a result, when a breach of hygiene protocol was observed during the delivery process, a midwife would wait for a more private time to point this out over immediate real-time correction. Conversely, because of this hierarchy, midwives felt no discomfort or hesitation in immediately correcting any visitors’ behaviours when noncompliance to hygiene practices was observed, publicly or otherwise.

## Discussion

This formative research study explored the hand hygiene practices and associated determinants of HCW during labour and delivery across eight health care facilities in Kampong Chhnang, Cambodia. We found that HCW hand hygiene compliance during uncomplicated vaginal births was low and hand hygiene worsened as the birth process progressed. Hand hygiene compliance rates did not differ by facility type or HCW qualification, but differed by HCW shift with adequate hand hygiene less likely to be observed during the night shift. The midwives’ hand hygiene practices were influenced by psychological, environmental and contextual factors including habits, norms, time, workload, inadequate knowledge and infection risk perception.

Previous hand hygiene studies in Cambodia have employed the use of self-reported behaviour and proxy measures of handwashing behaviour to assess HCW hand hygiene practices [26, 27, 44, 45]. Self-reporting is not recommended as a reliable method to assess handwashing behaviour due to over-reporting, and proxy measures do not accurately reflect the actual handwashing practice [46–48]. Our study adds to this existing literature by quantifying HCW hand practices using the recommended gold standard of direct observation [3, 49] as well as providing theoretically-informed determinants of the observed practices.

Our study findings are consistent with a recent systematic review of birth attendants’ hand hygiene compliance in health facilities in LMIC that estimated low compliance rates ranging between 1.3% and 38% [14]. Facility-based hand hygiene studies in Cambodia similarly found practices across all staff levels in various HCF departments [44, 45] and specifically among midwives during newborn post-natal care [26, 27] to be suboptimal and were influenced by a lack of adequate training, basic infrastructure and poor implementation of multimodal hand hygiene improvement strategies.

Hand hygiene in our settings was suboptimal even with ample physical opportunity and adequate knowledge specifically around hand hygiene protocol prior to the initiation of aseptic procedures in the absence of recontamination.. A recent systematic review found that physical opportunity (increasing access to infrastructure and materials) and capability (improving knowledge) are the most widely researched determinants of hygiene behaviours in birth environments and targeted by the majority of hand hygiene interventions [24]. Our findings, however, are consistent with other studies that show low compliance with hand hygiene protocol even in the presence of functioning infrastructure [17, 42, 50] or adequate levels of awareness/knowledge of hand hygiene practices and protocols among HCWs [51]. Interventions for improving hand hygiene among health care workers could be more responsive to the context-specific drivers of existing behaviours to address this gap in compliance.

Our study has identified several of these key context-specific determinants. Situations with increased time pressure and high workload were associated with breaches in hygiene protocol, similar to findings from other studies [52]. Low infection risk perception was a barrier to hand hygiene compliance related to potential recontamination of hands and to newborn aftercare. Additional training on opportunities for recontamination and the need to maintain hygiene protocol along the full continuum of care are possible avenues for addressing these determinants. However, increasing psychological capabilities e.g. knowledge alone, have shown to be ineffective in sustaining improved HCW hygiene behaviours [13, 51, 53] and comprehensive strategies targeting multiple behavioural determinants have been shown to be most effective [12, 13, 19].

Inappropriate use of gloves was a common cause of aseptic technique invalidation and inadequate hand hygiene among HCW in our study. Similar studies report frequent instances of glove misuse such as unnecessary multiple gloving [53, 54], inadequate and infrequent glove changing [17, 55], and no practice of hand hygiene during glove donning and glove changing [17, 39]. Similar to findings by Buxton et. al in delivery rooms in Nigeria, inadequate gloving practices in our study were employed as a timesaving hand hygiene substitute and midwives perceived these practices as safe, maintaining glove sterility and uncontaminated hands [53]. Midwives were unable to maintain compliance, commonly invalidating aseptic technique through recontamination of gloves from touching unclean surfaces or materials. Glove and hand recontamination before aseptic procedures has been described as a major barrier to maintaining compliance in labour wards in Tanzania [39], Nigeria [17] and Ghana [56]. Further research is needed to understand avoiding recontamination, its associated determinants and its specific role in HCW hand hygiene compliance [39, 57, 58].

Hand hygiene actions – including glove use – observed in our study were deeply embedded in existing routines and similar patterns were observed across observations. Glove changes, for example, were observed and reported to happen around the same points within the delivery flow every birth suggesting being cued by the sequence of procedures rather than by a hygiene or glove indication. Our findings show that uncomplicated births in this setting were often driven by habit—performed with little variation around who performed the delivery, where the care practices and associated hand hygiene opportunities occurred within the delivery room and when specific hygiene activities were conducted with the broader sequence of care. Facility based studies in well-resourced health systems have evaluated the important role of habit in determining HCW behaviours such as prescribing practices, examinations, providing referrals, etc. [59, 60]. The role of automatic processes in determining the HCW behaviours in resource constrained health care settings is understudied and warrants further investigation for the implementation of contextually appropriate interventions.

Habits are cued by context, therefore altering the context will cause a disruption of the existing routine practices and enable new behaviours to be inserted and instilled as routine [61]. Nudges and cues are a way to alter the physical or social environment within which the behaviour occurs and automatically trigger the performance of the desired behaviour [62]. Nudge- and cue-based handwashing interventions in LMIC have not been evaluated in health facility settings, however the limited evidence from school and humanitarian settings report positive effects of nudges on handwashing practices [63]. Handwashing increased by 64% in schools in Bangladesh following the application of a footpath nudge between the toilet and the handwashing facility [64] and in an internally displaced camp in Iraq, children who received a product-based nudge (toys embedded in a piece of soap) were four times more likely to wash their hands with soap than children who did not receive [63, 65]. Our study showed that hand hygiene opportunities were location-specific and linked with specific care practices. Conspicuous visual cues can be strategically placed in these locations to nudge adequate hand hygiene at the right times. Common areas such as PPE storage areas, the door, newborn aftercare area, delivery trolley, delivery bed and floor directly beneath it should be targeted.

The pre-existing strong social cohesion among the midwives can be leveraged to encourage the adoption of adequate practice at every hand hygiene indication as a norm. Individuals who feel strong affiliation to a group are more likely to adhere to the norms of that group [66]. The new norm could be promoted at a team level and integrated using existing multi modal strategies that target the social context of the midwives [19, 67]. Within Cambodia, the existing societal social and cultural hierarchies such as power relationships based on education level and gender, are often embedded within the hospital professional structures and have been highlighted as important influences of hygiene and overall care practices of the various HCF staff [68, 69]. Social influence components within hand hygiene improvement multimodal strategies have been directly associated with an improvement in HCW hand hygiene compliance in other hospital settings [70]. Approaches could include group pledging and making public commitments for behaviour adoption and norm setting [67], peer monitoring for accountability and self-regulation, and peer-to-peer evaluation and group feedback to provide opportunities for social comparison [71, 72].

Our study had some potential limitations. The sampling methodology of our study limits the generalisability of our findings to our study HCF and particularly to uncomplicated vaginal births of women presenting early at the facility. However, underpinning the study with a behavioural theory allows for some generalisable findings to be taken from this context. Participant reactivity may have led to an overestimate of hand hygiene compliance, despite masking the aim of the study during data collection to avoid any explicit mention of measuring hand hygiene compliance. Detailed transcripts of qualitative data were not prepared in English or Khmer. The BCD framework presented pre-defined categories against which data were compared and specific interview activities were targeted around a limited number of select determinants. As such, line-by-line coding of interviews / transcripts was not conducted. This pragmatic approach to qualitative analysis may have limited the nuance and depth of our exploration, but still allowed for the identification of broad patterns within the data and actionable hypotheses for further exploration and testing. Lastly, we did not collect information on hand washing techniques or duration and while this may not affect our study outcome, it may limit the effect on overall health impact (reduction of infection).

## Conclusion

The women and newborns in our study sites were at risk of infections associated with low levels of hand hygiene. Our study highlighted low levels of HCW hand hygiene compliance during labour and delivery and identified several factors influencing the observed practices. Using a theoretical approach, we were able to identify specific intervention targets, important drivers of behaviour and how to use the existing context to leverage behavioural change. Insights from formative research suggest that a multicomponent intervention targeted at midwives (and tailored to relevant determinants) may be an effective way to address the most important determinants and improve hand hygiene compliance among HCW.

## Supplementary Information

**Additional file 1.** Inclusion and Exclusion Criteria for structured observation.

**Additional file 2.** Detailed description of data collection tools used for formative research.

## Data Availability

The datasets used and/or analysed during this study are available from the corresponding author on reasonable request.

## Abbreviations

HCAI: Healthcare associated infections
HCF: Healthcare facilities
HCW: Healthcare workers
IPC: Infection prevention and control
MOH: Ministry of Health
WASH: Water, Sanitation and Hygiene
PHC: Primary health centres
RH: Referral hospital
BCD: Behaviour Change design
LMIC: Low- and middle-income countries
WHO: World Health Organisation
