# Supplementary material for: Hand hygiene during facility-based childbirth in Cambodia: a theory-driven, mixed-methods observational study
Source: BMC Pregnancy Childbirth. 2021 Jun 17;21:429. doi: 10.1186/s12884-021-03901-7 (PMC8212449; doi:10.1186/s12884-021-03901-7)
Supplement: Supplementary file 1 — Additional file 1. Inclusion and Exclusion Criteria for structured observation. [file 12884_2021_3901_MOESM1_ESM.docx]

HAND HYGIENE DURING FACILITY-BASED CHILDBIRTH IN CAMBODIA: A THEORY-DRIVEN, MIXED-METHODS OBSERVATIONAL STUDY

Yolisa Nalule^1, *^, Helen Buxton^2^, Por Ir^3^, Supheap Leang^3^, Alison Macintyre^4^, Ponnary Pors^5^, Channa Samol^5^, Robert Dreibelbis^1^

^1^ Disease Control Department, London School of Hygiene and Tropical Medicine, London WC1E 7HT, UK; [Yolisa.Nalule@lshtm.ac.uk](mailto:Yolisa.Nalule@lshtm.ac.uk) (YN); [Robert.Dreibelbis@lshtm.ac.k](mailto:Robert.Dreibelbis@lshtm.ac.k) (RD)

^2^ Division of Psychiatry, University College London, London, London W1T 7BN [helen.buxton.20@ucl.ac.uk](mailto:helen.buxton.20@ucl.ac.uk) (HB)

^3^ National Institute of Public Health, Phnom Penh, Cambodia; [ipor@niph.org.kh](mailto:ipor@niph.org.kh) (PI), [leangsupheap@yahoo.com](mailto:leangsupheap@yahoo.com) (SL)

^4^ WaterAid Australia, Melbourne, Australia; [alison.macintyre@unimelb.edu.au](mailto:alison.macintyre@unimelb.edu.au) (AM)

^5^ WaterAid Cambodia, Phnom Penh, Cambodia; [porsponnary@gmail.com](mailto:porsponnary@gmail.com) (PP), [samolchanna@yahoo.com](mailto:samolchanna@yahoo.com) (CS)

^*^  Correspondence: [Yolisa.Nalule@lshtm.ac.uk](mailto:Yolisa.Nalule@lshtm.ac.uk)

# ADDITIONAL INFORMATION

**Additional File 1: Inclusion and Exclusion Criteria for structured observation**

| Criteria | Patient (Woman) | Health care worker |
| --- | --- | --- |
| Inclusion |  |  |
|  | - Women in stage 1 active labour: cervical dilation >3cm (estimated by cervical examination performed by midwife/doctor)^1^ - Women admitted to deliver at the facility - Clinical staff present (midwife, nurse or doctor) - Provide consent for the study | - Works – either in a clinical or non-clinical capacity – in labour and delivery and/or post-natal care wards within the facility - Staff responsible for the cleaning or management of maternity or neonatal care areas (SSIs only) - Provide consent for the study |
| Exclusion | - Women who present in stage 2 labour: cervical dilation of 10cm (estimated by cervical examination performed by midwife/doctor); - Women who are in pain or distress prior to enrolment - Women with complicated labour, delivery or postnatal care - Women who are separated from the neonate after delivery due to complications - Absence of a clinical staff member during stage two or stage three labour - Women under the age of 18 years not accompanied by her partner or guardian^2^ | - Not involved in labour and delivery or post-natal care wards |

The primary rationale for this exclusion is an ethical concern related to whether it is appropriate to consent a women during active labour when she is likely to be in pain and/or distress. Agreement to participate commits the woman to up to 16 hours of observation, both at the health facility and at home, which is a significant undertaking. Any potential perceived coercion or perception that medical care is dependent on participation in the research study is much more likely to occur when the woman is in physical distress during labour.

Cervical examinations were not compulsory for the recruitment process. If an enrolled participant was <3 cm dilated following their first vaginal examination, the women would remain enrolled in the study but observations would not commence at that point. Observations would commence when the woman went into active labour as indicated by the birth attendants and confirmed by birth attendant at an appropriate moment in the woman’s care through cervical examination to monitor birth progress (>3cm dilated)*.* There could be more women enrolled in the study than were actually observed.

2 The Royal Government of Cambodia’s Law on Suppression of Human Trafficking and Sexual Exploitation (2006) define a minor as any individual under the age of 18. All protocols were reviewed and approved by ethical review committees in both Cambodia and the UK. While specific provisions may be in place that allow for enrolling pregnant minors into research studies, the research team decided to adhere to the legal age of majority for a number of reasons. Given the time at which women were enrolled (when presenting at the facility for childbirth), the researcher team was concerned that younger mothers (those below the age of 18) could feel compelled to participate in the research due to perceived power imbalances between the research team and the participant; the primary objectives of the research – understanding hand hygiene during the peri-natal period – could be achieved without the inclusion of pregnant minors.
