# Supplementary material for: Hand hygiene during facility-based childbirth in Cambodia: a theory-driven, mixed-methods observational study
Source: BMC Pregnancy Childbirth. 2021 Jun 17;21:429. doi: 10.1186/s12884-021-03901-7 (PMC8212449; doi:10.1186/s12884-021-03901-7)
Supplement: Supplementary file 2 — Additional file 2. Detailed description of data collection tools used for formative research. [file 12884_2021_3901_MOESM2_ESM.docx]

HAND HYGIENE DURING FACILITY-BASED CHILDBIRTH IN CAMBODIA: A THEORY-DRIVEN, MIXED-METHODS OBSERVATIONAL STUDY

Yolisa Nalule^1, *^, Helen Buxton^2^, Por Ir^3^, Supheap Leang^3^, Alison Macintyre^4^, Ponnary Pors^5^, Channa Samol^5^, Robert Dreibelbis^1^

^1^ Disease Control Department, London School of Hygiene and Tropical Medicine, London WC1E 7HT, UK; [Yolisa.Nalule@lshtm.ac.uk](mailto:Yolisa.Nalule@lshtm.ac.uk) (YN); [Robert.Dreibelbis@lshtm.ac.k](mailto:Robert.Dreibelbis@lshtm.ac.k) (RD)

^2^ Division of Psychiatry, University College London, London, London W1T 7BN [helen.buxton.20@ucl.ac.uk](mailto:helen.buxton.20@ucl.ac.uk) (HB)

^3^ National Institute of Public Health, Phnom Penh, Cambodia; [ipor@niph.org.kh](mailto:ipor@niph.org.kh) (PI), [leangsupheap@yahoo.com](mailto:leangsupheap@yahoo.com) (SL)

^4^ WaterAid Australia, Melbourne, Australia; alison.macintyre@unimelb.edu.au (AM)

^5^ WaterAid Cambodia, Phnom Penh, Cambodia; [porsponnary@gmail.com](mailto:porsponnary@gmail.com) (PP), [samolchanna@yahoo.com](mailto:samolchanna@yahoo.com) (CS)

^*^  Correspondence: [Yolisa.Nalule@lshtm.ac.uk](mailto:Yolisa.Nalule@lshtm.ac.uk)

**Additional File 2: Detailed description of data collection tools used for formative research**

### Semi-structured Interviews

Semi-structured interviews were conducted to gather self-reported barriers and motivators for handwashing and infection, prevention and control practices. Participants’ perceptions of cleanliness and hygiene, sources of information of IPC and handwashing practices and client satisfaction with quality of care were also gathered from this data.

As part of semi-structured interviews, each response had a set of guiding probes to delve deeper into behaviour determinants and challenges faced.

As the SSI data is all self-reported, the behaviour-focused BCD formative research tools (routine scripting and scenarios + ranking exercises) introduced within the context of the SSI functioned as elicitation methods to probe deeper into behaviour determinants and challenges, particularly non-conscious drivers, that may not easily be explained by the people involved. This avoided making assumptions about the role of planned, motivated and reactive behaviours in relation to handwashing and IPC behaviours based on what participants say about their behaviour.

### Routine Scripting

Routine Scripting was done with an aim of eliciting the normal, prototypical order of events in the everyday role of selected participants around specific areas/events with emphasis on practices related to the target behaviour. Each specific area of interest had a set of guiding questions to probe into behaviour determinants and barriers.

### Scenarios + Ranking

Using hypothetical scenarios (based on actual events observed during structured observations), this activity was meant to gain insight into the kinds of factors that constrain or promote performance of the target behaviour, while exploring participants’ attitudes or beliefs about particular situations. Using hypothetical scenarios provided an unthreatening and impersonal/indirect avenue for exploring certain topics that were vulnerable to the social desirability bias.

The scenario ranking exercise was aimed at gaining an insight into the relative importance of different hygiene breaches from the perspective of the respondent.
